# Taxonomic and functional surrogates of sessile benthic diversity in Mediterranean marine caves

**DOI:** 10.1371/journal.pone.0183707

**Published:** 2017-09-06

**Authors:** Vasilis Gerovasileiou, Charalampos Dimitriadis, Christos Arvanitidis, Eleni Voultsiadou

**Affiliations:** 1 Institute of Marine Biology, Biotechnology and Aquaculture, Hellenic Centre for Marine Research, Heraklion, Crete, Greece; 2 Department of Zoology, School of Biology, Aristotle University of Thessaloniki, Thessaloniki, Greece; 3 National Marine Park of Zakynthos, Zakynthos, Greece; University of Genova, ITALY

## Abstract

Hard substrates host globally a rich biodiversity, orders of magnitude higher in species number than that in surrounding soft substrates. Among them, marine caves support unique biodiversity and fragile communities but suffer lack of quantitative data on their structure and function, hindering their conservation status assessment. A first approach to the non-destructive ecological monitoring of marine caves by testing surrogates of structural and functional composition of sessile benthos was attempted in two species-rich Mediterranean marine caves. Photographic sampling was performed in different positions on the cave walls, across the horizontal axis, from the entrance inwards. Eighty-four taxa were identified and assigned to 6 biological traits and 32 modalities related to morphology, behavior and ecological affinities, with sponges being the dominant taxon in species richness and coverage. In quest of possible biological surrogates, we examined the spatial variability of the total community structure and function and separately the sponge community structure and function. The observed patterns of the above metrics were significantly correlated with the distance from the entrance, the small-scale variability and their interaction. A positive correlation was found between all examined pairs of those metrics, supporting that: (i) the developed functional approach could be used for the study of marine cave sessile communities, and (ii) sponges could be used as a surrogate taxon for the structural and functional study of these communities. The suggested method could be tested in other types of hard substrate habitats and in multiple locations of the Mediterranean waters, facilitating monitoring schemes and conservation actions.

## Introduction

The use of effective biological surrogates has proved to be a robust method for the study of community patterns, facilitating the conservation of marine biodiversity [1–7]. To date, the approach of biological surrogates has been tested in a wide variety of marine habitats but its effectiveness varies among different spatial scales, habitat types and methods used [8]. According to the latter global meta-analysis, surrogate effectiveness has been shown to be lower when habitat complexity increased (e.g. in coral reefs supporting high species and functional diversity).

Marine caves, which are a distinctive hard substrate habitat, have been characterized as “biodiversity reservoirs” of high conservation value, supporting unique and species-rich communities which harbor exclusive taxa and relict lineages at the global scale [9–11]. Although marine caves are widely distributed in rocky coasts of the world ocean, little research effort has been invested in comparison to other hard substrate habitat types. In karstic areas of the Mediterranean basin, marine caves form one of the most emblematic features, with at least 3000 caves penetrating the rocky coasts of this semi-enclosed sea [12].

Several human-induced pressures and threats along with environmental alterations have been identified as potentially detrimental for the marine cave communities [12–16]. Marine cave ecosystems present low recovery potential [17]. Due to their fragility, unique features, and biological wealth, marine caves are protected under the EU Habitats Directive (92/43/EEC, Habitat type 8330) and the Barcelona Convention [18–19]. However, quantitative historical data on the marine cave biota are lacking for most regions of the world, hindering the assessment of potential declines in habitat quality and subsequently the implementation of the relevant international and European legislation.

Marine cave communities have been studied in the Mediterranean more intensively than in any other area of the world ocean at spatial scales [10–11]. However, only a limited number of studies investigated quantitatively the community structure and diversity gradients of sessile benthos in marine caves focusing on the western and central Mediterranean sectors [17, 20–24]. During those studies, consensus has been reached that cave sessile communities are highly heterogeneous, reflecting the cave-specific topography, light intensity gradients, and hydrodynamic regime, with their distribution usually following a pattern of biotic impoverishment towards the aphotic end. In several cases, the small-scale spatial variability, for instance between opposite walls or sites within the same cave zone, can be higher than that between caves [25]. Besides the small-scale spatial variability, different facies may develop or be absent from marine caves located in different biogeographic regions and several invertebrate species have been recorded only in small numbers or even in a single cave [10]. Such differences highlight the need for studying communities in marine caves of diverse geomorphological types and different biogeographical regions.

Functioning of subterranean ecosystems is understudied and insufficiently understood at a global scale [26]. Relevant knowledge from marine caves is based mostly on description of the trophic structure indicating that the gradual decrease of diversity, coverage, and biomass of sessile taxa is accompanied by the dominance, decline or disappearance of certain taxa belonging to particular morphological and feeding groups; thus, large-sized suspension-feeders are replaced by small-sized encrusting filter-feeders which can cope with the increasing oligotrophy [14, 17, 27]. Aspects of trophic resource availability, such as carbon cycling and resource partitioning among groups have been rarely investigated in the marine cave system [28–31]. A theoretical model of structure and functioning of marine caves and an ecosystem-based approach was recently suggested for evaluating the ecological quality of caves in the western Mediterranean Sea [32].

Given the fragility of marine cave communities and the logistic constraints in underwater fieldwork in these dimly-lit or aphotic, space-limited environments, there is a critical need for developing non-destructive protocols for the study and monitoring of cave communities. The use of effective surrogate taxa could provide useful options for such approaches and further assist in marine cave conservation. Morphological diversity measures and standardized classification schemes, related to ecosystem functions, have been also suggested as a means for studying the structural and functional composition of sessile benthos in several hard habitat types, the marine caves included, enabling wide implementation even by non-taxonomists [33–37]. However, the functional diversity and composition of hard substrate sessile communities has been rarely examined, at least in the Mediterranean Sea, and the traits linked to the ecosystem functioning of sciaphilic communities have not yet been clearly identified [38].

Herein, we attempt to address the current need for evaluating and monitoring the marine cave communities. By investigating the structural and functional composition of sessile benthos in two marine caves with distinct morphology in an understudied Mediterranean ecoregion (the Aegean Sea), we examine the use of effective surrogate taxa. In this context, three main hypotheses have been tested: (a) Distance from the cave entrance and position in the cave (i.e. left wall, right wall, and ceiling) account for the observed spatial variability patterns of structural and functional benthic composition; (b) Gradients in the taxonomic composition of cave benthic communities are also reflected in their functional patterns; (c) Sponges, which are the dominant group in the studied caves, reflect the taxonomic and functional patterns of the entire macrobenthic community and thus they could be used as a surrogate taxon for the cave community structure and function. To our best knowledge, this is the first study that explores in such detail the functional structure of marine cave sessile communities.

## Materials and methods

### Study area

Two submerged marine caves of the eastern Mediterranean Sea were examined in the course of this study. The chosen caves are the best studied ones in the eastern Mediterranean and have been classified among the richest Mediterranean marine caves in terms of sponge diversity, exhibiting different diversity gradients [10, 36]. Both caves are rarely visited by SCUBA divers.

The caves are located on two islets off Lesvos Island (North Aegean, Eastern Mediterranean) and represent distinct morphological types and associated gradients of water confinement, according to Riedl’s [39] categorization: Agios Vasilios cave (38.969° N, 26.541° E) is a funnel-shaped, blind cave (16 m high x 7 m wide) with a large entrance and a depth range of 24–40 m, allowing light penetration for the first 15 m of this big, semi-dark cavern. The cave bed is covered by soft sediments and scattered boulders within the first 15 m from the entrance. Thereafter, it becomes rocky and is covered by a thin layer of silt at the inner dark part of the cave.

In this investigation, only the first 20 m part of the cave was studied, because beyond this distance, an ascending narrow and dark tunnel makes underwater work almost impossible.

Fara cave (38.969° N, 26.477° E is 32 m long tunnel, penetrating through the largest part of Fara islet and connecting to a smaller cave on the opposite side of the islet. The cave entrance is situated at a depth of 18 m, while the ceiling at its inner edge ascends to a depth of 11 m (the average depth of the cave interior is 14 m). The conical-shaped entrance (7 m high x 10 m wide) leads to a narrow corridor (only 2 m wide) resulting to a sharp decrease of light within the first 5–10 m. The inner chamber of the cave (20–32 m) is entirely dark. The cave bed is covered with soft silty sediment.

**Fig 1:**
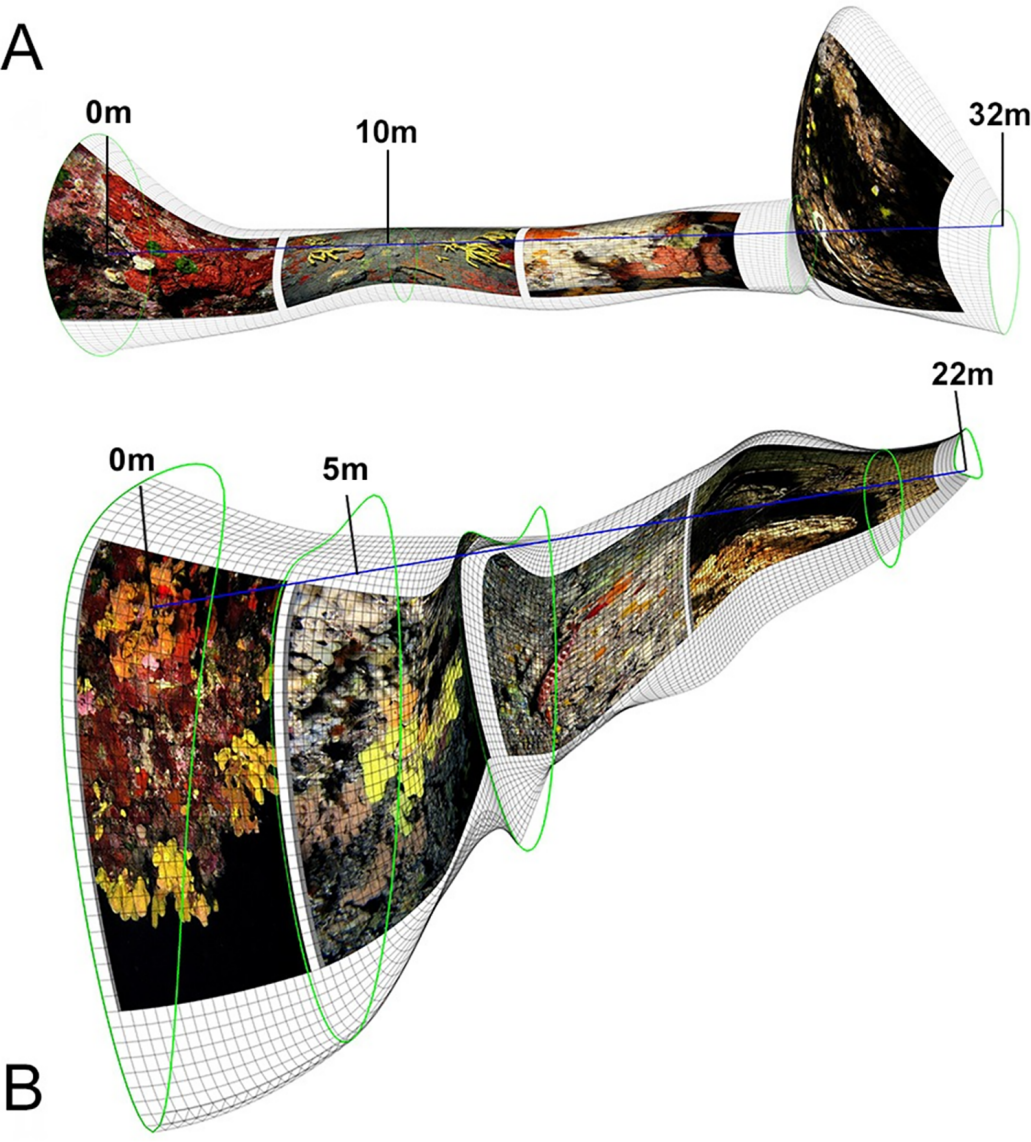
**Three-dimensional sliced models with representative images of different communities, distributed along the entrance-interior axis of (A) Fara and (B) Agios Vasilios caves, as visualized with ‘cavetopo’ software [40].** The blue lines represent the start-to-end cave axes along which distance measurements were taken. Green circles represent interpolated cross-sections of the caves, providing a three-dimensional perspective to the models.

Three-dimensional sliced models with representative images of different communities, distributed along the entrance-interior axis of the two caves, were produced by ‘cavetopo’ software [40] and are visualized in Fig 1.

### Sampling protocol and image processing

Non-destructive sampling was performed with SCUBA diving, using 625 cm^2^ photoquadrats. In each cave, three replicate quadrats were photographed at 5 m intervals, along the horizontal entrance-interior axis, on three transects: one along the cave ceiling (C) and two along the side walls (R: right and L: left). Due to the greater heterogeneity of organismic assemblages observed in the shallower conical entrance of Fara cave, quadrats were also photographed on the adjacent wall surfaces of the outer entrance zone (Out), which corresponded to a semi-sciaphilic community (zone II according to the zonation scheme suggested by Bianchi & Morri [27]. A total of 117 photoquadrats were taken in the two caves; 72 in Fara and 45 in Agios Vasilios corresponding to 45,000 and 28,125 cm^2^, respectively.

Additionally, a considerable number of qualitative samples were photographed *in situ* and collected from the two caves, to facilitate the identification of the sessile taxa observed in the photoquadrats.

To study the patterns of biological zonation, the photoquadrats were analyzed using photoQuad software, which is specialized for underwater ecological applications [41]. The external outline of each sessile taxon was defined manually using freehand drawing tools; then, defined areas of interest were assigned to the corresponding taxa according to the identifications of the qualitative samples. Percent coverage of every taxon was automatically calculated by the software.

### Biological traits

In order to describe community functioning, 6 traits related to species’ morphology, behavior and ecological affinities were chosen. Each trait has several modalities [see 42], to which all the examined taxa were assigned, based on information obtained from the relevant scientific literature [32, 38, 43–45], and online databases [42, 46]. Since definitions for traits and modalities relevant to the marine cave ecosystem are lacking, as do studies on the assignment of cave taxa to specific modalities, the 32 modalities adopted in the present study are presented in Table 1. Given that each taxon can exhibit multiple trait modalities to varying degrees, we adopted a “fuzzy coding” approach [47], according to which each taxon took a score for each exhibited modality ranging from 0 to 1, so that the overall score per trait always summed up to 1 [48]. The constructed “trait by taxon” matrix was then combined with the “coverage by photoquadrat” matrix (S1 File), thus producing the “trait by photoquadrat” matrix, on which all subsequent functional analysis was based [49]. To create this matrix, the coverage of each taxon at each photoquadrat was multiplied by its fuzzy coding score for each trait modality and then it was summed up across all taxa found at each photoquadrat [48, 50].

**Table 1:** Biological traits and trait modalities used for studying the functional structure in the surveyed marine caves.

Traits and modalities	Description
**Ecosystem engineering**	
Habitat formers	Providing habitat via their own living body
Constructors	Building structures via their mineral skeletons
Binders	Expanding and uniting the components of the habitat framework
Borers	Penetrating actively in hard substrata
Others	
**Maximum coverage**	
<0.3%	Max coverage in the quadrats lower than 0.3%
0.3–1%	Max coverage in the quadrats between 0.3 and 1%
1–3%	Max coverage in the quadrats between 1 and 3%
3–10%	Max coverage in the quadrats between 3 and 10%
10–30%	Max coverage in the quadrats between 10 and 30%
>30%	Max coverage in the quadrats greater than 30%
**Feeding type**	
Producers	Producing biomass from inorganic compounds
Suspension-feeders	Feeding on material suspended in the water
Filter-feeders	Actively filtering the water via their own water circulation system
**Morphology (body design)**	
Arborescent	Erect, branching habit, tree-like
Tubular	Shape of hollow, erect cylinder
Massive	Large, compact structure without definable shape
Encrusting	Thin, sheet-like coating of the substratum
Nodular	Forming nodules
Tunic	Tunic-shaped
Tube	Living in tubes
Shell	Shelled taxa
Calyx	In the form of a calyx
Foliaceous	In the form of a leaf
Filamentous	In the form of very thin threads or fibers
**Stratification**	
Endolithic layer	Living inside the hard substratum
Basal layer	Vertical growth up to 1 cm
Intermediate layer	Vertical growth between 1 and 10 cm
Upper layer	Vertical growth above 10 cm
**Sociability**	
Solitary	Living alone, not gregarious
Gregarious	Growing in clusters
Modular/Colonial	Comprising modules/closely associated conspecific individuals

### Structural and functional patterns

Two-way permutational multivariate analysis of variance (PERMANOVA) [51] was used to examine the effect of internal cave topography on: (i) total community structure–TCS (including all taxa encountered, using “taxa by photoquadrat” matrix), (ii) total community function–TCF (using “taxa by trait matrix”) and (iii) sponge community function–SCF (using “sponge taxa by trait” matrix) of each cave. PERMANOVA results on the effect of internal cave topography on the sponge community structure–SCS (including solely the sponge community) were derived from [36]. PERMANOVA was run on triangular similarity matrices derived from the fourth root transformed data calculating the Bray-Curtis coefficient, and considering two topographic factors (hypothesis a): (i) distance from entrance (Di), fixed with eight levels for Fara cave (Out, 0, 5, 10, 15, 20, 25, and 30 m) and five levels for Agios Vasilios cave (0, 5, 10, 15 and 20 m); (ii) position (Po), fixed with three levels for both caves (C: cave ceiling, L: left wall, R: right wall). The taxa and trait contribution to the observed Bray-Curtis dissimilarity with respect to the two topographic factors (Di and Po) was investigated by means of a two-way crossed SIMPER analysis [52]. Visualization of the observed patterns was obtained by means of non-metric MDS using the Bray-Curtis similarity matrix.

### Combined patterns—Surrogates

The relationship between TCS, TCF, SCS and SCF was investigated in order to examine whether the developed approach for studying TCF could be used as a surrogate for the study of TCS (hypothesis b) as well as the potential use of sponges as a surrogate taxon of studying the marine cave community at structural and functional levels (hypothesis c). To this end, the RELATE analysis [52], which enables comparative (Mantel-type) tests between triangular similarity matrices, was employed in order to investigate the strength and significance of the relationship between all the pairs of triangular similarity matrices representing the structural and functional multivariate patterns of the total community and sponge community separately (using weighted Spearman correlation index ρ_w_) for each cave. Other sessile groups were not taken into account separately in RELATE analysis, since they had small spatial coverage and patchy distributions across the cave zones.

All analyses were performed using PRIMER-E v6 [52] and PERMANOVA + software packages [53].

## Results

### Taxonomic composition

The analysis of photoquadrats revealed 84 taxa belonging to 9 major groups (S1 Table); the majority of the examined organisms were identified to the species or genus level (74 taxa), while the rest were assigned to higher categories, such as Serpulidae, Vermetidae, Hydrozoa, or to taxonomic/morphological groups such as turf-forming algae, encrusting Rhodophyta, non-calcified Bryozoa and encrusting Bryozoa. A total of 72 taxa were recorded in Fara cave and 64 taxa in Agios Vasilios cave, with 52 taxa being common to both caves.

### Substratum coverage

**Fig 2:**
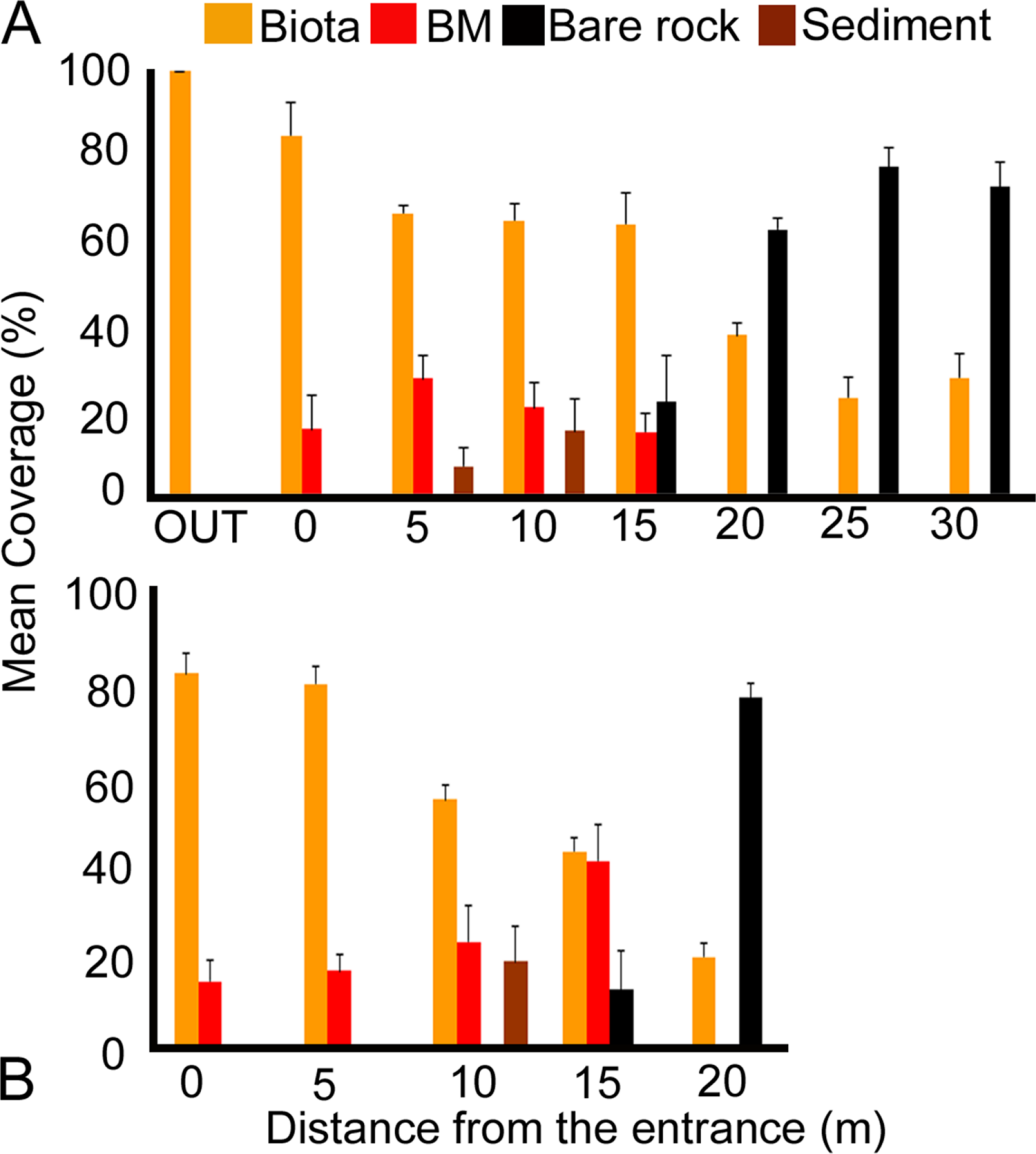
**Substratum coverage along the horizontal axis of (A) Fara cave and (B) Agios Vasilios cave.** Standard error of mean (SE) is presented in error bars. BM: unidentified non-living biogenic material.

A similar gradient in substratum coverage was observed in both caves (Fig 2), with the mean biotic coverage (including Serpulidae tubes) reaching from 80–100% of the substratum in the outer and entrance zones to 20–30% in the inner caves zones (lowest coverage was 10–11% at stations 25C of Fara cave and 20L of Agios Vasilios cave). The remaining surface was covered with unidentified, non-living biogenic material (e.g. dead rhodophytes, scleractinians, and bryozoans) within the first 15 m of the surveyed caves, and was replaced by bare rock inwards. In Fara cave, bare rock surfaces were covered with a thin black coating of Fe-Mn oxides, while in Agios Vasilios cave this was observed only in micro-fissures of the ceiling, at a distance of 10–15 m from the entrance. Particular areas on the side walls of the caves were covered with a layer of sediment (5L and 10 L in Fara cave; 10R and 10L in Agios Vasilios cave).

**Fig 3:**
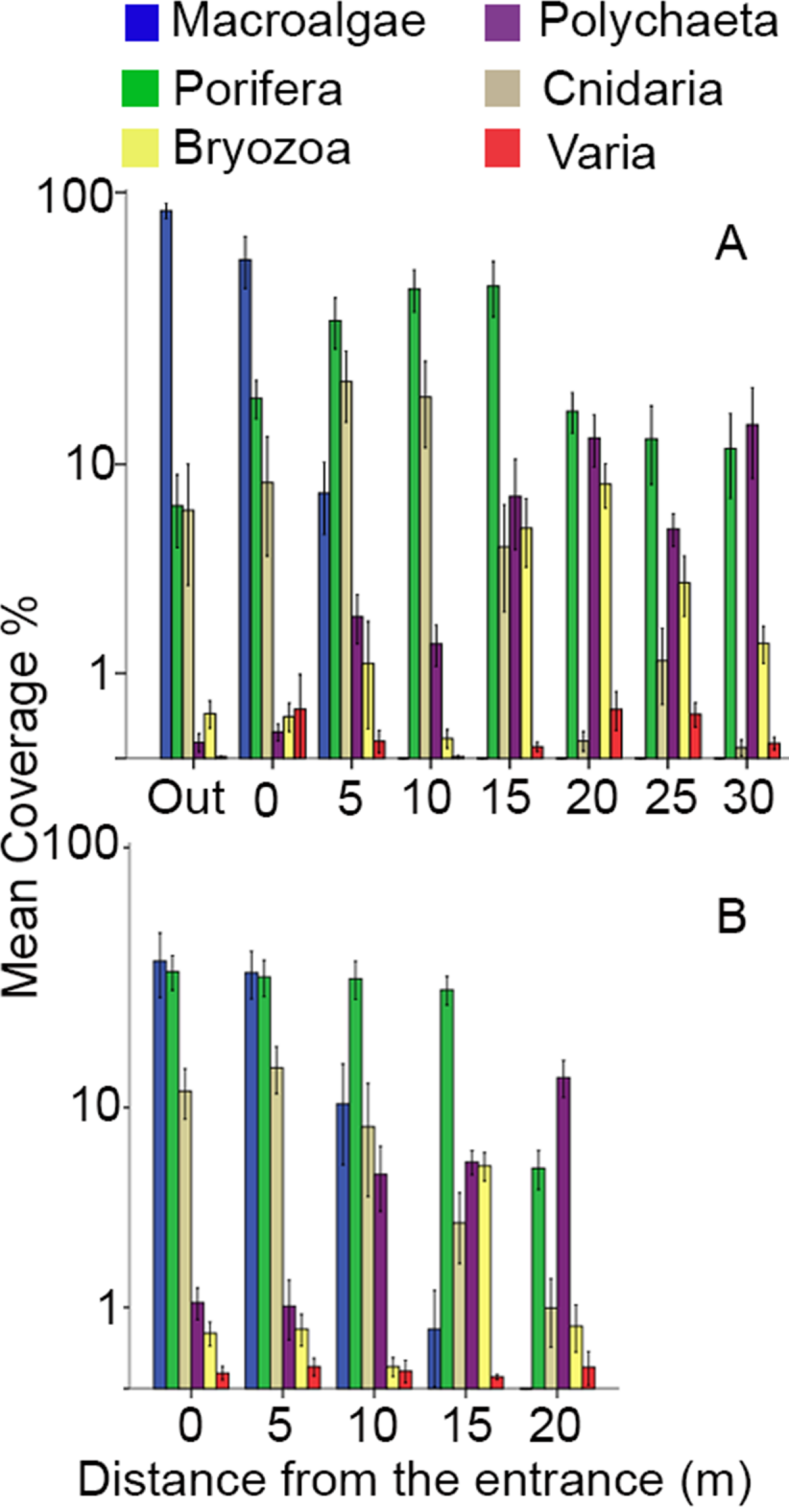
**Coverage per taxon (log scale) along the horizontal axis of (A) Fara cave and (B) Agios Vasilios cave.** Standard error of mean (SE) is presented in error bars. Varia includes Foraminifera, Mollusca, Brachiopoda and Ascidiacea.

Regarding total biotic coverage, Porifera dominated in both surveyed caves, followed by macroalgae, cnidarians, polychaetes, and bryozoans; however, different groups dominated in the various distances across the two caves (Fig 3). Small differences in the coverage of certain taxa were also observed between the side walls and the ceilings (S1 Fig). Macroalgae dominated at the entrance of Fara cave, while their coverage was similar to that of sponges at the entrance of the deeper Agios Vasilios cave. Sponges dominated in the intermediate and inner zones of both caves. Scleractinian corals (mostly facies of *Madracis pharensis*) presented higher coverage on the ceilings of most cave zones. Coverage of serpulid polychaetes increased towards the interior of both caves, with a maximum coverage reaching locally 23% in Fara (30L) and 17% in Agios Vasilios cave (20R). Bryozoans had small coverage values in most cave zones with the exception of the ceiling, at a distance of 15–20 m from the entrance in both caves, where a facies of nodule-forming encrusting bryozoans was recorded. The taxa Foraminifera, Mollusca, Brachiopoda, and Ascidiacea presented low coverage values.

### Dominant taxa and traits

Different taxa showed varying coverage in diverse parts of the caves (S1 Table). Encrusting rhodophytes presented the highest coverage in both caves, though their development was limited to the first 5 m of Fara cave and first 15 m of Agios Vasilios cave. The scleractinian *Madracis pharensis* and serpulid polychaetes followed, presenting a wide distribution throughout the two caves. The sponges *Spirastrella cunctatrix*, *Dendroxea lenis*, and *Hexadella pruvoti* had considerable coverage in both caves. The majority of taxa had a maximum coverage of 3–10%, while only 8 taxa reached coverage higher than 30% of the substratum (i.e. turf-forming algae, encrusting Rhodophyta, the sponges *Dendroxea lenis*, *Hexadella racovitzai*, *Phorbas tenacior*, *Spirastrella cunctatrix*, the scleractinian *Madracis pharensis*, and the serpulid polychaetes).

Ecosystem engineering taxa mostly comprised binders, constructors, and habitat formers (S2 Table). Encrusting filter-feeders (mainly sponges) dominated in terms of number of taxa in both caves. Only three species (the sponges *Agelas oroides*, *Aplysina aerophoba*, and *Axinella cannabina*) created an upper layer, while most taxa formed the basal and intermediate layers. Modular/colonial taxa dominated (64), followed by solitary ones (17), with only 6 taxa having a gregarious development.

### Heterogeneity of total community structure and function

**Fig 4:**
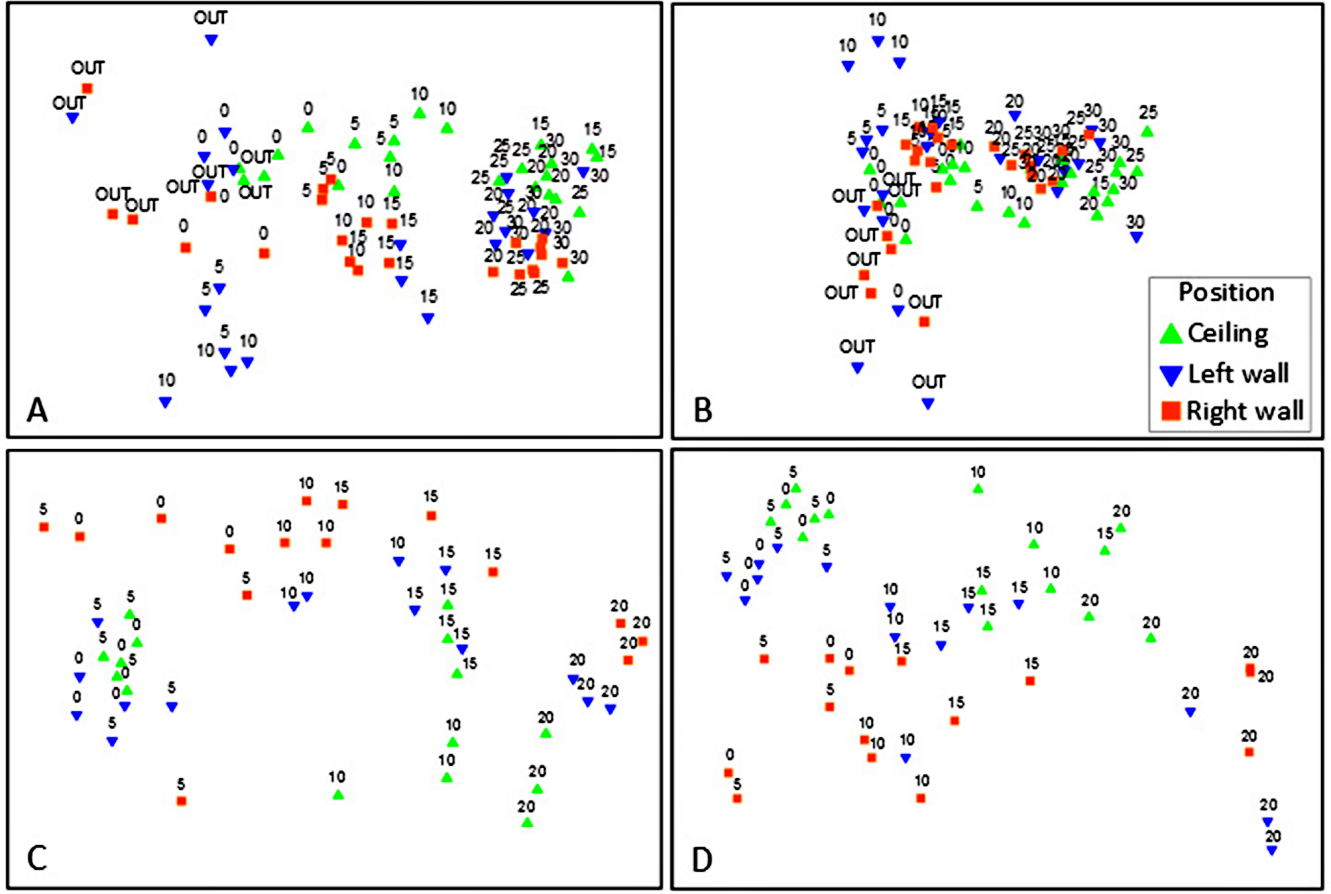
**Resemblance for (A) TCS and (B) TCF for Fara cave and (C) TCS and (D) TCF for Agios Vasilios cave with respect to the distance from the entrance and the position on the walls of the cave (ceiling, left wall, right wall).** Numbers indicate distance from cave entrance. TCS, total community structure; TCF, total community function.

Visualization of the patterns in the MDS plots suggested three major clusters of quadrats with respect to distance, for both TCS and TCF (Fig 4): (i) the first cluster comprised the anterior part of the caves (OUT-5 m in Fara cave and 0–5 m in Agios Vasilios cave); (ii) the second cluster comprised the intermediate part of the caves (10–15 m in both caves); and (iii) the third one comprised the innermost cave sector (20–30 m in Fara and 20 m in Agios Vasilios). Quadrats were also grouped according to their position on the cave walls, for both TCS and TCF, as depicted on the vertical axis of the MDS plots (Fig 4). In addition, quadrats from the ceiling of the entrance (0C) and intermediate zone (15C) of Fara cave were more similar to those of the intermediate and innermost sectors respectively.

According to the results of PERMANOVA, the distance from the entrance (Di), the position on cave walls (Po), and the interaction between these two factors significantly accounted for the observed pattern of TCS and TCF in both caves (Table 2). Pair-wise comparisons suggested that the members of all pairs of Di along the caves were significantly different from each other (*P* < 0.05 in all cases) regarding both TCS and TCF, with the exception of the pairs 25–30 m in Fara cave and 0–5 m in Agios Vasilios cave. As far as the factor Po is concerned, TCS and TCF were significantly different between the members of all pairs (*P* < 0.05 in all cases), except for the left and right walls of Fara cave, which emerged functionally similar. The greatest source of multivariate variability of TCS and TCF in both caves was Di, followed by individual quadrat level (i.e. Residuals), interaction of Di with Po, and Po in most cases (S3 Table). Two-way SIMPER analysis shows that the observed dissimilarity of TCS and TCF along the horizontal axis of both caves was mainly attributed to the successive replacement and coverage variation of different taxa and traits. At the vertical axis, particular taxa and traits were responsible for the observed dissimilarity of TCS and TCF throughout the caves (S4–S7 Tables).

**Table 2:** Results of PERMANOVA test with the distance and position factors for the total community structure and function in Fara and Agios Vasilios caves.

		Fara cave							Agios Vasilios cave					
		TCS			TCF				TCS			TCF		
**Source of Variation**	*df*	*MS*	*Pseudo—F*	*P*	*MS*	*Pseudo—F*	*P*	*df*	*MS*	*Pseudo—F*	*P*	*MS*	*Pseudo—F*	*P*
**Distance (Di)**	7	14070	17.978	0.001	2365.3	20.571	0.001	4	13307	24.582	0.001	1795.2	31.116	0.001
**Position (Po)**	2	6165.6	7.8782	0.001	1526.4	13.275	0.001	2	4384.8	8.1002	0.001	882.81	15.302	0.001
**Di x Po**	14	3052.1	3.8999	0.001	357.82	3.,112	0.001	8	1498.3	2.7679	0.001	221.75	3.8437	0.001
**Residuals**	48	782.62			114.9			30	541.32			57.693		
**Total**	71							44						

TCS, total community structure; TCF, total community function.

### Heterogeneity of sponge community structure and function

**Fig 5:**
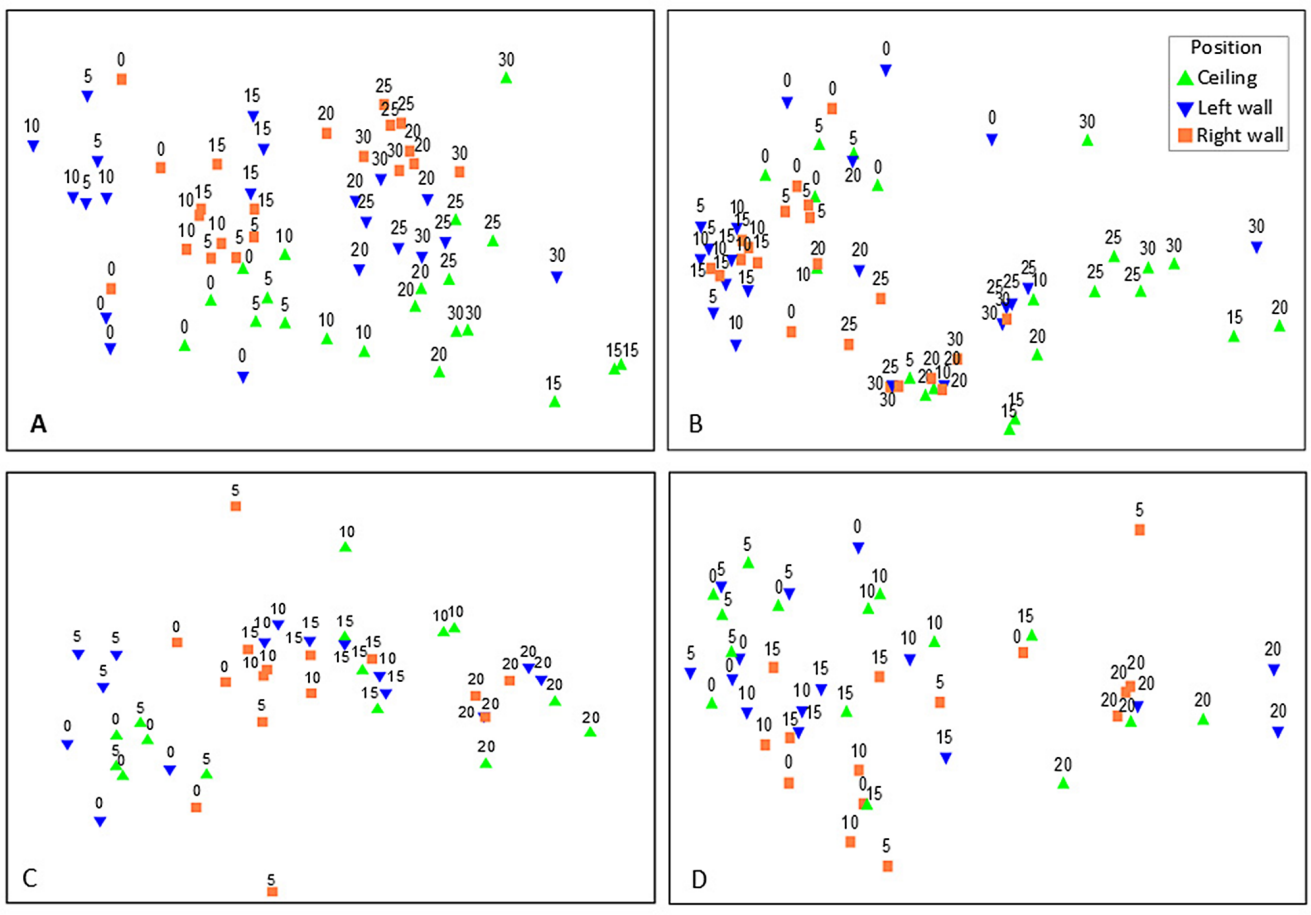
**Resemblance for (A) SCS and (B) SCF for Fara cave and (C) SCS and (D) SCF Agios Vasilios cave with respect to the distance from the entrance and the position on the walls of the cave (ceiling, left wall, right wall).** Numbers indicate distance from cave entrance. SCS, sponge community structure; SCF, sponge community function.

Similarly to TCS and TCF, a significant variability in the SCS and SCF was observed with respect to the factors Di, Po, and their interaction in both caves (Table 3, Fig 5). However, the greatest multivariate variability was observed at the scale of individual quadrats (i.e. Residuals) in Fara cave in contrast to Agios Vasilios cave where the most significant source of variability was Di (S3 Table). Regarding SCS, pair-wise comparisons showed that the members of all pairs differed significantly regarding Di with the exception of the pairs 5–10 m and 25–30 m in Fara and the pair 5–10 m in Agios Vasilios cave. Regarding the factor Po, both caves presented significant pair-wise differences between the members of all pairs. Considering SCF, pair-wise comparisons in Fara cave revealed significant differences along Di, apart from the outermost and innermost cave sectors (pairs 0*–*5 m and 25*–*30 m), whereas in Agios Vasilios cave, significant differences were detected only when the innermost cave sector (Di = 20 m) was compared with the rest of the cave. The functional structure of the ceiling was significantly different in comparison to the side walls of both caves. Similarly to TCS and TCF, the successive replacement and coverage variation of sponge taxa and traits was responsible for the dissimilarity across the horizontal and vertical cave axes (S8–S11 Tables for Two-way crossed SIMPER results).

**Table 3:** Results of PERMANOVA test with the distance and position factors for the sponge community structure and function in Fara and Agios Vasilios caves.

		Fara cave							Agios Vasilios cave					
		SCS			SCF				SCS			SCF		
**Source of Variation**	*df*	*MS*	*Pseudo—F*	*P*	*MS*	*Pseudo—F*	*P*	*df*	*MS*	*Pseudo—F*	*P*		*MS*	*Pseudo—F*	*P*
**Distance (Di)**	7	12233	9.0503	0.001	2564.2	4.8777	0.001	4	2335.6	21.211	0.001	15816	18.586	0.001
**Position (Po)**	2	7159.3	5.2966	0.001	2568.6	4.8861	0.001	2	509.82	4.63	0.003	3197.4	3.7574	0.001
**Di x Po**	14	4381.6	3.2415	0.001	892.12	1.697	0.001	8	362.21	3.289	0.001	1819.7	2.1384	0.001
**Residuals**	48	1351.7			525.7			30	110.11			850.96		
**Total**	71							44						

SCS, sponge community structure; SCF, sponge community function.

### Combined patterns—Surrogates

The results of the RELATE test showed that all examined pairs of TCS, TCF, SCS and SCF presented significant positive correlation with one another (Table 4). Specifically, the strongest correlation was detected between TCS and SCS. Correlation of TCS with TCF was the second highest, followed by the correlation of TCF with SCF. Both caves exhibited similar patterns of correlation with respect to the rank of the compared pairs according to the strength of their relationship (Spearman rank correlation coefficient values).

**Table 4:** Strength and significance of the relationship (RELATE test) between all pairs of similarity matrices regarding the structural and functional patterns of the total community and the sponge community of Fara and Agios Vasilios caves.

	Fara cave		Agios Vasilios cave	
	Rho	*P*	Rho	*P*
TCS *vs*. SCS	0.826	0.001	0.882	0.001
TCS *vs*. TCF	0.807	0.001	0.777	0.001
TCF *vs*. SCF	0.760	0.001	0.687	0.001
SCS *vs*. TCF	0.717	0.001	0.645	0.001
SCS *vs*. SCF	0.642	0.001	0.574	0.001
TCS *vs*. SCF	0.460	0.001	0.494	0.001

TCS, total community structure; SCS, sponge community structure; TCF, total community function; SCF, sponge community function.

## Discussion

Sessile communities on hard substrate bottoms exhibit characteristic patterns of vertical zonation, reflecting environmental gradients [54–56]. Specifically, hard substrate communities in marine caves are characterized by a marked horizontal zonation, even within the scale of few meters [17, 27, 39, 57]. However, the lack of quantitative data describing the community structure in marine caves has been highlighted as a major impediment for the assessment of potential declines in the habitat quality through time [15, 58]. This study provides the first quantitative baseline of the current structural and functional composition of sessile hard-substrate benthos in marine caves with distinct morphology, in an ecoregion understudied for its cave biota, the Aegean Sea. This analysis is crucial for caves frequently visited by SCUBA divers [see 59], caves exposed to coastal and marine infrastructure activities [16] and for those located in areas subjected to global warming and biological invasions, such as the eastern and southern Mediterranean [13–15, 60]. Moreover, as most Mediterranean marine caves studied for their biota are located in the shallow littoral zone of the north-western basin [10], this study extends the current knowledge eastwards and to the lower sublittoral zone.

### Gradients in structure and function of cave communities

The results of this study confirm the hypothesis that topographic factors, such as distance from the cave entrance, position on cave walls, and their interaction, account for the observed patterns of spatial variability for both the total and sponge community structure and both the total and sponge community function in both caves. Distance from the cave entrance emerges as the most significant factor shaping these variability patterns by inducing a strong environmental gradient of light availability and water confinement across the cave system [17, 27, 61]. The results show, however, that small-scale variability (i.e. quadrat level and position on cave walls) also contributes considerably to the observed patterns; in particular, the quadrat level variability occurs to be more prominent than that of distance for the sponge community structure and function in Fara cave. The unique geomorphology among distinct caves and different zones within the same cave has been suggested to induce small-scale heterogeneity of benthic communities [25].

The study of community structure in the surveyed caves reveals three main benthic communities: a coralligenous community, limited to the outer and entrance zones of the two caves, a semi-dark cave community in the intermediate sectors, and a dark cave community in the inner cave zones, following the general bionomic scheme described by Pérès [57] for the Mediterranean Sea. In this context, there was a shift in taxa composition along the cave axes (both horizontal and vertical), and a strong coverage variation for species widely distributed in the cave system (e.g. *Spirastrella cunctatrix*, *Agelas oroides*, *Madracis pharensis*, *Dendroxea lenis*, and Serpulidae). Increasing oligotrophy, reflected by the decrease of biotic coverage and biomass, has been considered as one of the critical features shaping community gradients in the marine cave habitat [17, 27]. In the surveyed caves, biotic coverage decreases from 100% of the substratum at the entrance to only 10% at the inner dark sectors with the vast majority of taxa reaching a maximum coverage not higher than 3–10% and forming a basal/intermediate level of stratification.

At the community functioning scale, in most cases, trait modalities are represented by several species with variable coverage in the different cave sectors, probably indicating that the marine cave system sustains a high degree of functional redundancy [62]. Thus, functional redundancy may safeguard cave communities against the strong environmental gradient by maintaining important ecosystem processes [63]. Similar results have been also reported from other types of transitional ecosystems and environmental gradients [64].

The horizontal penetration of macroalgae and their specific trait modalities regarding feeding type (i.e. producers) and body design (i.e. foliaceous and filamentous) towards the intermediate walls of Agios Vasilios cave (0–15 m) is most probably related to the broader dimensions of the cave entrance (16 m x 7 m) and the subsequent difference in light regime, compared to that of Fara cave (7 m x 10 m) where macroalgae disappeared within the first 5 m.

The low productivity in groundwater environments severely affects trophic specialization of cave taxa [65]. The elimination of light with increasing depth, distance from the entrance, and changes in substratum inclination in the examined caves contributes to the progressive disappearance of producers and the dominance of space-competing modular/colonial animals [23, 39, 66].

The community structure and function differed among the cave walls, even inside zones. Thus, in both caves, photoquadrats from the ceiling of the entrance (0C) and intermediate cave zones (15C) were more similar to those of the inner semi-dark and dark sectors respectively than to the quadrats of the same zones, probably due to the sharper decrease of light at the ceilings than at the corresponding side walls, as a result of the substratum inclination [27, 67]. Accordingly, the coverage of macroalgae was lesser on the shadowed ceiling than on the side walls of the cave entrance. The development of suspension-feeding, calyx-shaped anthozoans forming dense clusters (e.g. *Madracis pharensis* and *Leptopsammia pruvoti*) on the ceilings can be attributed not only to the dim-light conditions and subsequent elimination of space-competing macroalgae, but also to the turbulent water movement at the ceiling level, supplying scleractinians with additional organic particles [68].

According to the pattern of biotic stratification on marine cave walls described for the north-western Mediterranean [32], an upper layer of sessile invertebrates is often created by suspension-feeding anthozoans (e.g. gorgonians and the red coral *Corallium rubrum*), the latter being usually limited to specific cave zones (i.e. entrance and ceiling) as they directly depend on active water flow for nutrition; some of these species are used as refuges by macro-invertebrates [69]. These cnidarians are absent or rare in marine caves of the eastern Mediterranean Sea [70], where the upper biotic layer may be formed by massive/tubular and arborescent sponges, as shown in the results of the present study. In the studied caves, this layer was not limited to the entrance zone, since sponges are active water filterers. Arborescent *Axinella* species were found on the left wall of Fara cave 5 to 10 m from the entrance; in this zone, hard substratum is covered by a thick sediment layer, reflecting a locally higher sedimentation rate, probably due to the positive wall inclination and its proximity to the silty cave bed. Arborescent sponges can better cope with increased sedimentation as their body design helps to prevent the clogging of their canals [71]. The development of arborescent sponges in silty cave sectors has been documented in the Balearic Islands [72] and witnessed in several caves of the Aegean Sea (VG unpublished data).

An interesting biotic component in marine caves is the habitat-forming sponges, which may be rare and small-sized in marine caves of the western Mediterranean basin, but are found in considerably large sizes and massive/tubular forms in caves of the eastern basin (this study, VG unpublished data). These sponges can support a rich associated macrofaunal assemblage and maintain their functional role as ecosystem engineers across the caves by increasing habitat complexity, this role becoming particularly important in the dark cave sectors [73].

Borers, on the other hand, often form a considerable proportion of benthic biomass in marine caves [66]. In the studied caves, borers (i.e. sponges of the genus *Cliona* and the bivalves *Lithophaga lithophaga* and *Rocellaria dubia*) created an endolithic layer mainly in the anterior part of the caves, probably due to the increased presence of constructors in this zone (e.g. rhodophytes and scleractinians), which produce the easily perforable calcareous substratum, as suggested in previous studies [74].

Interestingly, the spatial patterns observed for the cave community function were highly correlated to those of community structure and influenced by the same factors (hypothesis b). This fact implies that changes in species composition due to human induced stressors and climate change effect would subsequently influence the functioning of the sessile benthos, thus increasing the vulnerability of the already fragile cave system [75]. In this respect, the conservation of marine caves is of high importance.

Across the cave gradient, a strong environmental filtering of ecological traits of the biota was observed since species with similar traits co-occur and functionally resemble each other in their need to respond to the same factors. On the other hand, competitive interactions among taxa may possibly account for the observed community structure and trait distribution at a smaller spatial scale (local effect) since particular trait modalities were responsible for the observed functional dissimilarity within the cave sectors and positions. This fact probably denotes that at local scale, or at the microhabitat level, species might compete with each other due to the limited resources that the cave environment provides (e.g. space close to the entrance, light availability and trophic resources inwards).

### Surrogates of cave sessile community structure and function

The use of particular taxa as surrogates of a chosen community should be based on their effectiveness in achieving conservation objectives [2, 7]. However, surrogate effectiveness highly depends on the complexity of the examined habitat [8]. Despite their varying degree of natural heterogeneity, marine caves are characterized by a decrease of structural complexity with regards to their sessile communities towards the impoverished dark interior [17]. Our hypothesis that sponges, being the dominant phylum in the studied caves, could also be used as a surrogate taxon for the study of sessile community structure and function was also confirmed.

The use of dominant taxa as surrogates for the rapid assessment of spatial diversity patterns has been to date suggested mainly for soft substratum macrobenthos [e.g. 1, 76]. Concerning hard substratum communities, the morphological diversity of sponges has been suggested as a surrogate measure in studies of cave sponge diversity gradients by non-experts [36]. Moreover, sponges have been recently used as indicators of habitat quality in a small number of citizen science initiatives, e.g. in tropical coral reefs [37] and Mediterranean coralligenous formations [77]. The results of the present study confirmed the significant functional role of sponges, as a keystone taxon [see 2], for the marine cave habitat since the sponge functional pattern was highly correlated with the one derived from the analysis of the total community function, further highlighting the potential use of this taxon as an indicator for the monitoring of this particular habitat. Nevertheless, further testing is needed in order to evaluate the efficiency of sponges as surrogates of the cave sessile community structure and function in different Mediterranean areas, especially in caves where other invertebrate taxa (e.g. arborescent cnidarians) interplay with sponges in terms of spatial coverage and biomass. The approach developed in the course of this work for studying the community function of marine caves does not aim to substitute proper taxonomic screening of sessile benthic species which forms a prerequisite for baseline biodiversity studies and effective monitoring schemes. However, it enables the easy assignment of sessile taxa to basic functional traits and modalities, assisting in the development of non-destructive rapid assessment methods, able to indicate abrupt alterations in this particular environment by using much less information and taxonomic expertise. The suggested methodology could be tested in other types of hard substrates as well, considering the particular species composition, in order to assist ecological assessment, conservation and management.

## Supporting information

S1 File Coverage by photoquadrat and trait by taxon data matrices. (XLSX)

S1 Table Taxa recorded and their mean percent coverage (Co), distribution (Di) along the horizontal axis, and position on the walls (Po) of the surveyed caves. OUT, outer zone; 0–30, distance from entrance in meters; C, cave ceiling; L. left wall; R, right wall. Data on Porifera are according to [36].
(PDF)

S2 Table Number of taxa (N) per trait and modality and their distribution (Di) along the horizontal axis, and position on the walls (Po) of the surveyed caves. OUT, outer zone; 0–30, distance from entrance in meters; C, cave ceiling; L. left wall; R, right wall.
(PDF)

S3 Table Ranking (1–4) of the measured sources of variation of PERMANOVA test results in the studied caves. Di, distance from entrance; Po, position on the cave walls; TCS, total community structure; TCF, total community function; SCS, sponge community structure; SCF, sponge community function.
(PDF)

S4 Table **Summary of (A) taxa and (B) traits contributing by 50% to the calculated Bray-Curtis dissimilarity between the successive pairs of distance levels (5 m intervals) along the horizontal axis of Fara cave (Two-way crossed SIMPER analysis results), indicated with grey color.** ns, non-significant difference according to the results of PERMANOVA. For abbreviations of modalities see S2 Table.
(PDF)

S5 Table **Summary of (A) taxa and (B) traits contributing by 50% to the calculated Bray-Curtis dissimilarity between the different positions in Fara cave (Two-way crossed SIMPER analysis results), indicated with grey color.** C, cave ceiling; L, left wall; R, right wall; ns, non-significant difference according to the results of PERMANOVA. For abbreviations of modalities see S2 Table.
(PDF)

S6 Table **Summary of (A) taxa and (B) traits contributing by 50% to the calculated Bray-Curtis dissimilarity between the successive pairs of distance levels (5 m intervals) along the horizontal axis of Agios Vasilios cave (Two-way crossed SIMPER analysis results), indicated with grey color.** For abbreviations of modalities see S2 Table.
(PDF)

S7 Table **Summary of (A) taxa and (B) traits contributing by 50% to the calculated Bray-Curtis dissimilarity between the different positions in Agios Vasilios cave (Two-way crossed SIMPER analysis results), indicated with grey color.** C, cave ceiling; L, left wall; R, right wall. For abbreviations of modalities see S2 Table.
(PDF)

S8 Table **Summary of sponge (A) taxa and (B) traits contributing by 50% to the calculated Bray-Curtis dissimilarity between the successive pairs of distance levels (5 m intervals) along the horizontal axis of Fara cave (Two-way crossed SIMPER analysis results), indicated with grey color.** ns, non-significant difference according to the results of PERMANOVA. For abbreviations of modalities see S2 Table.
(PDF)

S9 Table **Summary of sponge (A) taxa and (B) traits contributing by 50% to the calculated Bray-Curtis dissimilarity between the different positions in Fara cave (Two-way crossed SIMPER analysis results), indicated with grey color.** C, cave ceiling; L, left wall; R, right wall; ns, non-significant difference according to the results of PERMANOVA. For abbreviations of modalities see S2 Table.
(PDF)

S10 Table **Summary of sponge (A) taxa and (B) traits contributing by 50% to the calculated Bray-Curtis dissimilarity between the successive pairs of distance levels (5 m intervals) along the horizontal axis of Agios Vasilios cave (Two-way crossed SIMPER analysis results), indicated with grey color.** ns, non-significant difference according to the results of PERMANOVA. For abbreviations of modalities see S2 Table.
(PDF)

S11 Table **Summary of sponge (A) taxa and (B) traits contributing by 50% to the calculated Bray-Curtis dissimilarity between the different positions in Agios Vasilios cave (Two-way crossed SIMPER analysis results), indicated with grey color.** C, cave ceiling; L, left wall; R, right wall; ns, non-significant difference according to the results of PERMANOVA. For abbreviations of modalities see S2 Table.
(PDF)

S1 Fig Coverage per taxon along the horizontal axis of the ceiling, left wall and right wall of Fara and Agios Vasilios caves. Standard error of mean (SE) is presented in error bars.
(TIF)
