# Supplementary material for: Taxonomic and functional surrogates of sessile benthic diversity in Mediterranean marine caves
Source: PLoS One. 2017 Sep 6;12(9):e0183707. doi: 10.1371/journal.pone.0183707 (PMC5587111; doi:10.1371/journal.pone.0183707)
Supplement: S1 Table — OUT, outer zone; 0–30, distance from entrance in meters; C, cave ceiling; L. left wall; R, right wall. Data on Porifera are according to [36]. (PDF) [file pone.0183707.s002.pdf]

**S1 Table. Taxa recorded and their mean percent coverage (Co), distribution (Di) along the horizontal axis, and position on the walls (Po) of the surveyed caves.** OUT, outer zone; 0-30, distance from entrance in meters; C, cave ceiling; L. left wall; R, right wall. Data on Porifera are according to [36].

| Taxa | Fara cave | | | Agios Vasilios cave | | |
|---|---|---|---|---|---|---|
| | Co (%) | Di | Po | Co (%) | Di | Po |
| **Macroalgae** | | | | | | |
| *Codium bursa* (Olivi) C.Agardh | 0.2 | OUT | R | | | |
| Encrusting Calcareous Rhodophyta | 16.6 | OUT–5 | C, L, R | 14.3 | 0–15 | C, L, R |
| *Flabellia petiolata* (Turra) Nizamuddin | <0.1 | OUT | L | | | |
| *Palmophyllum crassum* (Naccari) Rabenhorst | 0.6 | OUT–5 | C, L, R | 0.6 | 0–10 | C, L, R |
| Turf-forming algae | 1.5 | OUT–5 | C, L, R | 1.1 | 0–5 | C, L, R |
| **Foraminifera** | | | | | | |
| *Miniacina miniacea* (Pallas, 1766) | <0.1 | 5–15 | C, L, R | <0.1 | 0–15 | C, L, R |
| **Porifera** | | | | | | |
| *Aaptos aaptos* (Schmidt, 1864) | <0.1 | 5 | C | | | |
| *Acanthella acuta* Schmidt, 1862 | 0.4 | OUT–10 | L, R | | | |
| *Agelas oroides* (Schmidt, 1864) | 3.4 | OUT–20 | C, L, R | 0.2 | 0–10 | C, L |
| *Aplysilla rosea* (Barrois, 1876) | <0.1 | OUT–5 | C, L | <0.1 | 0–15 | C, L, R |
| *Aplysina aerophoba* Nardo, 1833 | | | | 1.7 | 0–15 | C, L, R |
| *Axinella cannabina* (Esper, 1794) | 0.1 | 5–10 | L | | | |
| *Axinella damicornis* (Esper, 1794) | 0.4 | OUT–10 | L, R | 0.2 | 0–15 | C, L, R |
| *Axinella verrucosa* (Esper, 1794) | 0.2 | 5–10 | L | | | |
| *Clathrina* sp. | 0.1 | 0–30 | C, L, R | <0.1 | 15 | C, L |
| *Cliona celata* Grant, 1826 | <0.1 | OUT–10 | C, L, R | | | |
| *Cliona schmidti* (Ridley, 1881) | <0.1 | OUT–5 | C, L, R | <0.1 | 0 | C |
| *Cliona viridis* (Schmidt, 1862) | 0.1 | OUT–0 | C, L | | | |
| *Crella* sp. | | | | 0.1 | 15 | R |
| *Dendroxea lenis* (Topsent, 1892) | 4.3 | 0–30 | C, L, R | 3.2 | 0–15 | C, L, R |
| *Dictyonella incisa* (Schmidt, 1880) | 0.1 | OUT–10 | L, R | <0.1 | 15 | R |
| *Diplastrella bistellata* (Schmidt, 1862) | 1.4 | 15–30 | C, L, R | | | |
| *Dysidea fragilis* (Montagu, 1814) | 0.1 | 10 | L | | | |
| *Eurypon clavatum* (Bowerbank, 1866) | <0.1 | 15 | C | <0.1 | 15–20 | C |
| *Eurypon* sp. | 0.4 | 10–30 | C, L, R | 1.4 | 0–20 | C, L, R |
| *Fasciospongia cavernosa* (Schmidt, 1862) | <0.1 | OUT–5 | C, L | <0.1 | 5 | C |
| *Haliclona (Halichoclona) fulva* (Topsent, 1893) | 0.1 | 15–30 | L, R | <0.1 | 0 | L |
| *Haliclona (Soestella) mucosa* (Griessinger, 1971) | 0.4 | OUT–30 | C, L, R | 0.2 | 0–10 | C, L, R |
| *Haliclona* sp. | <0.1 | 0 | R | | | |
| *Hexadella pruvoti* Topsent, 1896 | 0.1 | OUT–30 | C, L, R | 1.7 | 0–15 | C, L, R |
| *Hexadella racovitzai* Topsent, 1896 | <0.1 | 10 | C, R | 2.7 | 0–15 | C, L, R |
| *Hexadella* sp. | 0.1 | 10–25 | C, L, R | <0.1 | 15 | L |
| *Ircinia oros* (Schmidt, 1864) | 0.1 | 0 | L | | | |
| *Ircinia paucifilamentosa* Vacelet, 1961 | 0.6 | 15–25 | L, R | 0.2 | 0–10 | C, L |
| *Ircinia variabilis* (Schmidt, 1862) | 0.2 | 10 | R | | | |
| *Jaspis johnstonii* (Schmidt, 1862) | 0.3 | 5–25 | L, R | | | |
| *Myrmekioderma spelaeum* (Pulitzer-Finali, 1983) | <0.1 | 30 | C | | | |
| *Oscarella balibaloi* Pérez et al., 2011 | <0.1 | 0–5 | C, R | 0.7 | 0–5 | L, R |
| *Oscarella microlobata* Muricy et al., 1996 | 0.2 | 20–30 | C, L, R | <0.1 | 15 | C |
| *Oscarella tuberculata* (Schmidt, 1868) | <0.1 | 10 | R | 1.6 | 0–15 | C, L, R |
| *Penares euastrum* (Schmidt, 1868) | 0.6 | 5–15 | L, R | 0.8 | 0–15 | C, L, R |
| *Petrosia (Petrosia) ficiformis* (Poiret, 1789) | 0.3 | OUT–15 | C, L, R | | | |
| *Phorbas tenacior* (Topsent, 1925) | 3.3 | OUT–15 | C, L, R | 1.1 | 0–5 | C, L, R |

| Taxa | Fara cave | | | Agios Vasilios cave | | |
|---|---|---|---|---|---|---|
| | **Co (%)** | **Di** | **Po** | **Co (%)** | **Di** | **Po** |
| *Plakina bowerbanki* (Sarà, 1960) | 0.4 | 20–30 | C, L, R | 3.6 | 0–20 | C, L, R |
| *Plakina* sp. | 0.2 | 0–30 | C, L, R | | | |
| *Plakina trilopha* Schulze, 1880 | | | | 0.6 | 0–20 | C, L, R |
| *Pleraplysilla spinifera* (Schulze, 1879) | 0.1 | OUT–15 | C, L, R | 0.4 | 0–15 | C, L, R |
| *Pseudocorticium jarrei* Boury-Esnault et al., 1995 | 0.1 | 0–10 | C, R | <0.1 | 0–5 | L, R |
| *Raspaciona aculeata* (Johnston, 1842) | 0.2 | 5–15 | C, L, R | 0.7 | 0–15 | C, L, R |
| *Sarcotragus foetidus* Schmidt, 1862 | | | | <0.1 | 5 | C |
| *Spirastrella cunctatrix* Schmidt, 1868 | 3.8 | OUT–15 | C, L, R | 3.7 | 0–10 | C, L, R |
| *Spongia (Spongia) virgultosa* (Schmidt, 1868) | <0.1 | OUT–0 | R | | | |
| *Terpios gelatinosa* (Bowerbank, 1866) | <0.1 | OUT–15 | L, R | <0.1 | 0–15 | R |
| *Tethya aurantium* (Pallas, 1766) | 0.1 | 0 | R | | | |
| *Thymosiopsis cuticulatus* Vacelet & Perez, 1998 | 0.3 | 5–15 | L, R | | | |
| *Timea unistellata* (Topsent, 1892) | <0.1 | 15 | R | 1.6 | 0–20 | C, L, R |
| **Cnidaria** | | | | | | |
| Hydrozoa sp. | <0.1 | OUT–5 | C | <0.1 | 0–5 | C |
| *Caryophyllia (Caryophyllia) inornata* (Duncan, 1878) | <0.1 | OUT–20 | C, L, R | <0.1 | 0–20 | C, L, R |
| *Ceratotrochus magnaghii* Cecchini, 1914 | <0.1 | 25 | C | <0.1 | 20 | C |
| *Hoplangia durotrix* Gosse, 1860 | 0.1 | OUT–30 | C, L, R | 1.3 | 0–20 | C, L, R |
| *Leptopsammia pruvoti* Lacaze-Duthiers, 1897 | 0.1 | OUT–30 | C, L, R | 1.1 | 0–20 | C, L, R |
| *Madracis pharensis* (Heller, 1868) | 7.2 | OUT–30 | C, L, R | 5.2 | 0–10 | C, L, R |
| *Paracyathus pulchellus* (Philippi, 1842) | <0.1 | 10–30 | C, L | <0.1 | 15–20 | C, L |
| *Phyllangia americana mouchezii* (Lacaze-Duthiers, 1897) | <0.1 | 5–10 | C, R | | | |
| **Polychaeta** | | | | | | |
| Serpulidae | 5.5 | OUT–30 | C, L, R | 5.3 | 0–20 | C, L, R |
| **Mollusca** | | | | | | |
| *Bryopa melitensis* (Broderip, 1834) | <0.1 | 5 | L | <0.1 | 0 | C |
| *Rocellaria dubia* (Pennant, 1777) | <0.1 | OUT | C | <0.1 | 0–5 | C, L |
| *Lithophaga lithophaga* (Linnaeus, 1758) | | | | <0.1 | 0–15 | C |
| Ostreoida sp. | <0.1 | 5 | R | <0.1 | 10 | C |
| Vermetidae | | | | <0.1 | 0–15 | C, R |
| **Brachiopoda** | | | | | | |
| *Novocrania anomala* (Müller, 1776) | 0.1 | 10–30 | C, L, R | <0.1 | 20 | C |
| Other Brachiopoda | 0.1 | 0–30 | C, L, R | <0.1 | 0–20 | C, L |
| **Bryozoa** | | | | | | |
| *Adeonella pallasii* (Heller, 1867) | 0.1 | 0–5 | R | <0.1 | 0–10 | C, R |
| Encrusting Bryozoa | 2.4 | 0–30 | C, L, R | 1.3 | 0–20 | C, L, R |
| *Frondipora verrucosa* (Lamouroux, 1821) | | | | <0.1 | 0–10 | R |
| Non-calcified Bryozoa | <0.1 | OUT–10 | C, R | 0.1 | 0–5 | C, L, R |
| *Rhynchozoon neapolitanum* Gautier, 1962 | 0.1 | OUT–5 | C, L, R | 0.1 | 0–10 | C, L, R |
| *Schizomavella* sp. | | | | <0.1 | 0 | R |
| *Reteporella* sp. | | | | <0.1 | 0 | R |
| *Turbicellepora coronopus* (Wood, 1844) | <0.1 | 0–5 | L | <0.1 | 0–5 | R |
| **Ascidiacea** | | | | | | |
| *Halocynthia papillosa* (Linnaeus, 1767) | | | | <0.1 | 0 | C, L |
| *Microcosmus* sp. | 0.1 | 0 | R | | | |
| Ascidiacea sp.1 | | | | <0.1 | 20 | C |
| Ascidiacea sp.2 | | | | 0.1 | 0–15 | C, L, R |
