# Supplementary material for: Taxonomic and functional surrogates of sessile benthic diversity in Mediterranean marine caves
Source: PLoS One. 2017 Sep 6;12(9):e0183707. doi: 10.1371/journal.pone.0183707 (PMC5587111; doi:10.1371/journal.pone.0183707)
Supplement: S2 Table — OUT, outer zone; 0–30, distance from entrance in meters; C, cave ceiling; L. left wall; R, right wall. (PDF) [file pone.0183707.s003.pdf]

S2 Table. Number of taxa (N) per trait and modality and their distribution (Di) along the horizontal axis, and position on the walls (Po) of the surveyed caves. OUT, outer zone; 0-30, distance from entrance in meters; C, cave ceiling; L. left wall; R, right wall.

**Functional traits and modalities**	**Abbreviation**	**Fara cave**			**Agios Vasilios cave**			**Both caves**
		**N**	**Di**	**Po**	**N**	**Di**	**Po**	**N**
**Ecosystem engineering**								
Habitat formers	Ec-Hf	13	OUT-25	C, L, R	13	0-20	C, L, R	18
Constructors	Ec-Co	18	OUT-30	C, L, R	21	0-20	C, L, R	22
Binders	Ec-Bi	35	OUT-30	C, L, R	29	0-20	C, L, R	37
Borers	Ec-Bo	4	OUT-10	C, L, R	3	0-15	C, L	5
Others	Ec-Ot	3	OUT-0	L, R	0	-	-	3
**Maximum coverage**								
<0.3%		9	OUT-25	C, L, R	12	0-20	C, L, R	14
0.3-1%		8	OUT-30	C, L, R	9	0-20	C, L, R	11
1-3%		10	OUT-30	C, L, R	9	0-20	C, L, R	12
3-10%		21	OUT-30	C, L, R	12	0-20	C, L, R	22
10-30%		16	OUT-30	C, L, R	12	0-20	C, L, R	17
>30%		8	OUT-30	C, L, R	8	0-20	C, L, R	8
**Feeding type**								
Producers	Ft-Pr	5	OUT-5	C, L, R	3	0-15	C, L, R	5
Suspension-feeders	Ft-Sf	9	OUT-30	C, L, R	8	0-20	C, L, R	9
Filter-feeders	Ft-Ff	58	OUT-30	C, L, R	51	0-20	C, L, R	70
**Morphology (body shape)**								
Arborescent	Mo-Ar	5	OUT-15	C, L, R	5	0-15	C, L, R	7
Tubular	Mo-Tu	2	OUT-20	C, L, R	2	0-15	C, L, R	3
Massive	Mo-Ma	12	OUT-25	C, L, R	6	0-15	C, L, R	13
Encrusting	Mo-En	36	OUT-30	C, L, R	29	0-20	C, L, R	38
Nodular	Mo-Nod	4	OUT-30	C, L, R	5	0-20	C, L, R	5
Tunic	Mo-Tun	1	0	R	3	0-20	C, L, R	4
Tube	Mo-Tub	1	OUT-30	C, L, R	2	0-20	C, L, R	2
Shell	Mo-Sh	5	OUT-30	C, L, R	6	0-20	C, L	6
Calyx	Mo-Ca	6	OUT-30	C, L, R	5	0-20	C, L, R	6
Foliaceous	Mo-Fo	1	OUT	L	0	-	-	1
Filamentous	Mo-Fi	2	OUT-10	C, L, R	2	0-5	C, L, R	2
**Stratification**								
Endolithic layer	St-En	5	OUT-10	C, L, R	4	0-15	C, L	6
Basal layer	St-Ba	44	OUT-30	C, L, R	37	0-20	C, L, R	46
Intermediate layer	St-In	29	OUT-30	C, L, R	25	0-20	C, L, R	37
Upper layer	St-Up	2	OUT-20	C, L, R	2	0-15	C, L, R	3
**Sociability**								
Solitary	So-So	12	OUT-30	C, L, R	15	0-20	C, L, R	17
Gregarious	So-Gr	6	OUT-30	C, L, R	5	0-20	C, L, R	6
Colonial/Modular	So-M/C	57	OUT-30	C, L, R	45	0-20	C, L, R	64
