# Supplementary material for: Taxonomic and functional surrogates of sessile benthic diversity in Mediterranean marine caves
Source: PLoS One. 2017 Sep 6;12(9):e0183707. doi: 10.1371/journal.pone.0183707 (PMC5587111; doi:10.1371/journal.pone.0183707)
Supplement: S3 Table — Di, distance from entrance; Po, position on the cave walls; TCS, total community structure; TCF, total community function; SCS, sponge community structure; SCF, sponge community function. (PDF) [file pone.0183707.s004.pdf]

S3 Table. Ranking (1-4) of the measured sources of variation of PERMANOVA test results in the studied caves. Di, distance from entrance; Po, position on the cave walls; TCS, total community structure; TCF, total community function; SCS, sponge community structure; SCF, sponge community function.

Source of Variation	Fara cave				Agios Vasilios cave			
	TCS	TCF	SCS	SCF	TCS	TCF	SCS	SCF
**Di**	1	1	2	2	1	1	1	1
**Po**	4	4	4	4	4	3	4	4
**Di x Po**	3	3	3	3	3	4	3	3
**Residuals**	2	2	1	1	2	2	2	2
