# Supplementary material for: Taxonomic and functional surrogates of sessile benthic diversity in Mediterranean marine caves
Source: PLoS One. 2017 Sep 6;12(9):e0183707. doi: 10.1371/journal.pone.0183707 (PMC5587111; doi:10.1371/journal.pone.0183707)
Supplement: S5 Table — Summary of (A) taxa and (B) traits contributing by 50% to the calculated Bray-Curtis dissimilarity between the different positions in Fara cave (Two-way crossed SIMPER analysis results), indicated with grey color. C, cave ceiling; L, left wall; R, right wall; ns, non-significant difference according to the results of PERMANOVA. For abbreviations of modalities see S2 Table. (PDF) [file pone.0183707.s006.pdf]

S5 Table. Summary of (A) taxa and (B) traits contributing by 50% to the calculated Bray-Curtis dissimilarity between the different positions in Fara cave (Two-way crossed SIMPER analysis results), indicated with grey color. C, cave ceiling; L, left wall; R, right wall; ns, non-significant difference according to the results of PERMANOVA. For abbreviations of modalities see S2 Table.

**(A) Total community structure**				**(B) Total community function**				
**Taxa**	**C vs L**	**C vs R**	**L vs R**	**Traits**	**Modalities**	**C vs L**	**C vs R**	**L vs R**
Madracis pharensis				Ecosystem engineering	Ec-Hf			ns
Dendroxea lenis					Ec-Co			ns
Spirastrella cunctatrix					Ec-Bi			ns
Agelas oroides				Maximum coverage	3-10%			ns
Hexadella pruvoti				Feeding type	Ft-Sf			ns
Serpulidae				Morphology (body design)	Mo-Nod			ns
Phorbas tenacior					Mo-Ma			ns
Haliclona mucosa					Mo-Tub			ns
Eurypon sp.					Mo-Tu			ns
Diplastrella bistellata					Mo-En			ns
Leptopsammia pruvoti				Stratification	St-Ba			ns
Encrusting Bryozoa					St-Up			ns
Axinella damicornis								
Acanthella acuta								
Turf-forming algae								
