# Supplementary material for: Taxonomic and functional surrogates of sessile benthic diversity in Mediterranean marine caves
Source: PLoS One. 2017 Sep 6;12(9):e0183707. doi: 10.1371/journal.pone.0183707 (PMC5587111; doi:10.1371/journal.pone.0183707)
Supplement: S6 Table — Summary of (A) taxa and (B) traits contributing by 50% to the calculated Bray-Curtis dissimilarity between the successive pairs of distance levels (5 m intervals) along the horizontal axis of Agios Vasilios cave (Two-way crossed SIMPER analysis results), indicated with grey color. For abbreviations of modalities see S2 Table. (PDF) [file pone.0183707.s007.pdf]

S6 Table. Summary of (A) taxa and (B) traits contributing by 50% to the calculated Bray-Curtis dissimilarity between the successive pairs of distance levels (5 m intervals) along the horizontal axis of Agios Vasilios cave (Two-way crossed SIMPER analysis results), indicated with grey color. For abbreviations of modalities see S2 Table.

(A) Total community structure

Taxa	0 vs 5	5 vs 10	10 vs 15	15 vs 20
Turf-forming algae	ns			
Spirastrella cunctatrix	ns			
Palmophyllum crassum	ns			
Aplysina aerophoba	ns			
Hoplangia durotrix	ns			
Hexadella racovitzai	ns			
Madracis pharensis	ns			
Penares euastrum	ns			
Encrusting Rhodophyta	ns			
Dendroxea lenis	ns			
Plakina bowerbankii	ns			
Timea unistellata	ns			
Raspaciona aculeata	ns			
Plakina trilopha	ns			
Encrusting Bryozoa	ns			
Hexadella pruvoti	ns			

(B) Total community function

Traits	Modalities	0 vs 5	5 vs 10	10 vs 15	15 vs 20
Ecosystem engineering	Ec-Hf				
	Ec-Bi				
Maximum coverage	0.3-1%				
	10-30%				
	1-3%				
	>30%				
	<0.3%				
	3-10%				
	Ft-Pr				
	Ft-Ff				
	Ft-Sf				
	Mo-Fi				
	Mo-Nod				
	Mo-Ca				
	Mo-Ma				
	Mo-Tun				
	Mo-Tu				
	Mo-En				
	Mo-Tub				
Stratification	St-Up				
	St-Ba				
Sociability	So-M/C				
