# Supplementary material for: Taxonomic and functional surrogates of sessile benthic diversity in Mediterranean marine caves
Source: PLoS One. 2017 Sep 6;12(9):e0183707. doi: 10.1371/journal.pone.0183707 (PMC5587111; doi:10.1371/journal.pone.0183707)
Supplement: S7 Table — Summary of (A) taxa and (B) traits contributing by 50% to the calculated Bray-Curtis dissimilarity between the different positions in Agios Vasilios cave (Two-way crossed SIMPER analysis results), indicated with grey color. C, cave ceiling; L, left wall; R, right wall. For abbreviations of modalities see S2 Table. (PDF) [file pone.0183707.s008.pdf]

S7 Table. Summary of (A) taxa and (B) traits contributing by 50% to the calculated Bray-Curtis dissimilarity between the different positions in Agios Vasilios cave (Two-way crossed SIMPER analysis results), indicated with grey color. C, cave ceiling; L, left wall; R, right wall. For abbreviations of modalities see S2 Table.

<table>
<tr><th colspan="4">(A) Total community structure</th><th colspan="5">(B) Total community function</th></tr>
<tr><th>Taxa</th><th>C vs L</th><th>C vs R</th><th>L vs R</th><th>Traits</th><th>Modalities</th><th>C vs L</th><th>C vs R</th><th>L vs R</th></tr>
<tr><td>Dendroxea lenis</td><td style="background:#808080"></td><td></td><td></td><td>Ecosystem engineering</td><td>Ec-Hf</td><td style="background:#808080"></td><td style="background:#808080"></td><td style="background:#808080"></td></tr>
<tr><td>Madracis pharensis</td><td style="background:#808080"></td><td></td><td></td><td>Maximum coverage</td><td>1-3%</td><td style="background:#808080"></td><td style="background:#808080"></td><td></td></tr>
<tr><td>Hexadella pruvoti</td><td style="background:#808080"></td><td></td><td></td><td></td><td>0.3-1%</td><td style="background:#808080"></td><td style="background:#808080"></td><td style="background:#808080"></td></tr>
<tr><td>Caryophyllia inornata</td><td style="background:#808080"></td><td></td><td></td><td></td><td>10-30%</td><td style="background:#808080"></td><td></td><td></td></tr>
<tr><td>Encrusting Bryozoa</td><td></td><td style="background:#808080"></td><td style="background:#808080"></td><td>Feeding type</td><td>Ft-Sf</td><td style="background:#808080"></td><td style="background:#808080"></td><td style="background:#808080"></td></tr>
<tr><td>Aplysina aerophoba</td><td></td><td style="background:#808080"></td><td style="background:#808080"></td><td></td><td>Ft-Ff</td><td></td><td></td><td style="background:#808080"></td></tr>
<tr><td>Spirastrella cunctatrix</td><td></td><td style="background:#808080"></td><td style="background:#808080"></td><td></td><td>Ft-Pr</td><td style="background:#808080"></td><td style="background:#808080"></td><td style="background:#808080"></td></tr>
<tr><td>Hexadella racovitzai</td><td></td><td style="background:#808080"></td><td style="background:#808080"></td><td>Morphology (body design)</td><td>Mo-Tu</td><td></td><td style="background:#808080"></td><td style="background:#808080"></td></tr>
<tr><td>Plakina trilopha</td><td></td><td style="background:#808080"></td><td style="background:#808080"></td><td></td><td>Mo-Ma</td><td></td><td style="background:#808080"></td><td style="background:#808080"></td></tr>
<tr><td>Serpulidae</td><td style="background:#808080"></td><td></td><td style="background:#808080"></td><td></td><td>Mo-Nod</td><td style="background:#808080"></td><td style="background:#808080"></td><td style="background:#808080"></td></tr>
<tr><td>Eurypon sp.</td><td style="background:#808080"></td><td></td><td style="background:#808080"></td><td></td><td>Mo-Ca</td><td style="background:#808080"></td><td style="background:#808080"></td><td style="background:#808080"></td></tr>
<tr><td>Encrusting Rhodophyta</td><td style="background:#808080"></td><td style="background:#808080"></td><td></td><td></td><td>Mo-En</td><td style="background:#808080"></td><td></td><td></td></tr>
<tr><td>Plakina bowerbankii</td><td style="background:#808080"></td><td style="background:#808080"></td><td></td><td>Stratification</td><td>St-Ba</td><td style="background:#808080"></td><td></td><td></td></tr>
<tr><td>Raspaciona aculeata</td><td style="background:#808080"></td><td style="background:#808080"></td><td></td><td></td><td>St-Up</td><td></td><td style="background:#808080"></td><td style="background:#808080"></td></tr>
<tr><td>Timea unistellata</td><td style="background:#808080"></td><td style="background:#808080"></td><td style="background:#808080"></td><td></td><td></td><td></td><td></td><td></td></tr>
<tr><td>Hoplangia durotrix</td><td style="background:#808080"></td><td style="background:#808080"></td><td style="background:#808080"></td><td></td><td></td><td></td><td></td><td></td></tr>
<tr><td>Penares euastrum</td><td style="background:#808080"></td><td style="background:#808080"></td><td style="background:#808080"></td><td></td><td></td><td></td><td></td><td></td></tr>
<tr><td>Leptopsammia pruvoti</td><td style="background:#808080"></td><td style="background:#808080"></td><td style="background:#808080"></td><td></td><td></td><td></td><td></td><td></td></tr>
<tr><td>Oscarella tuberculata</td><td style="background:#808080"></td><td style="background:#808080"></td><td style="background:#808080"></td><td></td><td></td><td></td><td></td><td></td></tr>
</table>
