# Supplementary material for: Taxonomic and functional surrogates of sessile benthic diversity in Mediterranean marine caves
Source: PLoS One. 2017 Sep 6;12(9):e0183707. doi: 10.1371/journal.pone.0183707 (PMC5587111; doi:10.1371/journal.pone.0183707)
Supplement: S9 Table — Summary of sponge (A) taxa and (B) traits contributing by 50% to the calculated Bray-Curtis dissimilarity between the different positions in Fara cave (Two-way crossed SIMPER analysis results), indicated with grey color. C, cave ceiling; L, left wall; R, right wall; ns, non-significant difference according to the results of PERMANOVA. For abbreviations of modalities see S2 Table. (PDF) [file pone.0183707.s010.pdf]

S9 Table. Summary of sponge (A) taxa and (B) traits contributing by 50% to the calculated Bray-Curtis dissimilarity between the different positions in Fara cave (Two-way crossed SIMPER analysis results), indicated with grey color. C, cave ceiling; L, left wall; R, right wall; ns, non-significant difference according to the results of PERMANOVA. For abbreviations of modalities see S2 Table.

**(A) Sponge community structure**				**(B) Sponge community function**				
**Taxa**	**C vs L**	**C vs R**	**L vs R**	**Traits**	**Modalities**	**C vs L**	**C vs R**	**L vs R**
Axinella damicornis			■	Ecosystem engineering	Ec-Hf	■		
Acanthella acuta			■		Ec-Bi	■	■	■
Diplastrella bistellata		■	■	Maximum coverage	>30%	■	■	■
Haliclona mucosa	■		■		10-30%		■	
Dendroxea lenis	■	■	■		3-10%			■
Agelas oroides	■	■	■	Feeding type	Ft-Ff	■	■	■
Spirastrella cunctatrix	■	■	■	Morphology (body design)	Mo-En		■	■
Hexadella pruvoti	■	■	■		Mo-Ma	■		
Phorbas tenacior	■	■	■	Stratification	St-Ba		■	■
					St-In	■		
				Sociability	So-M/C	■	■	■
