# Supplementary material for: Taxonomic and functional surrogates of sessile benthic diversity in Mediterranean marine caves
Source: PLoS One. 2017 Sep 6;12(9):e0183707. doi: 10.1371/journal.pone.0183707 (PMC5587111; doi:10.1371/journal.pone.0183707)
Supplement: S10 Table — Summary of sponge (A) taxa and (B) traits contributing by 50% to the calculated Bray-Curtis dissimilarity between the successive pairs of distance levels (5 m intervals) along the horizontal axis of Agios Vasilios cave (Two-way crossed SIMPER analysis results), indicated with grey color. ns, non-significant difference according to the results of PERMANOVA. For abbreviations of modalities see S2 Table. (PDF) [file pone.0183707.s011.pdf]

S10 Table. Summary of sponge (A) taxa and (B) traits contributing by 50% to the calculated Bray-Curtis dissimilarity between the successive pairs of distance levels (5 m intervals) along the horizontal axis of Agios Vasilios cave (Two-way crossed SIMPER analysis results), indicated with grey color. ns, non-significant difference according to the results of PERMANOVA. For abbreviations of modalities see S2 Table.

(A) Sponge community structure

Taxa	0 vs 5	5 vs 10	10 vs 15	15 vs 20
Oscarella tuberculata		ns		
Oscarella balibaloi		ns		
Phorbas tenacior		ns		
Spirastrella cunctatrix		ns		
Dendroxea lenis		ns		
Hexadella racovitzai		ns		
Plakina trilopha		ns		
Hexadella pruvoti		ns		
Penares euastrum		ns		
Raspaciona aculeata		ns		
Eurypon sp.		ns		
Timea unistellata		ns		
Plakina bowerbankii		ns		

(B) Sponge community function

Traits	Modalities	0 vs 5	5 vs 10	10 vs 15	15 vs 20
Ecosystem engineering	Ec-Hf				ns
	Ec-Bi				ns
Maximum coverage	>30%				ns
	10-30%				ns
	3-10%				ns
	1-3%				ns
Morphology (body design)	Mo-En				ns
	Mo-Tu				ns
	Mo-Ma				ns
Stratification	St-Up				ns
	St-Ba				ns
	St-In				ns
