# Supplementary material for: Taxonomic and functional surrogates of sessile benthic diversity in Mediterranean marine caves
Source: PLoS One. 2017 Sep 6;12(9):e0183707. doi: 10.1371/journal.pone.0183707 (PMC5587111; doi:10.1371/journal.pone.0183707)
Supplement: S11 Table — Summary of sponge (A) taxa and (B) traits contributing by 50% to the calculated Bray-Curtis dissimilarity between the different positions in Agios Vasilios cave (Two-way crossed SIMPER analysis results), indicated with grey color. C, cave ceiling; L, left wall; R, right wall; ns, non-significant difference according to the results of PERMANOVA. For abbreviations of modalities see S2 Table. (PDF) [file pone.0183707.s012.pdf]

**S11 Table. Summary of sponge (A) taxa and (B) traits contributing by 50% to the calculated Bray-Curtis dissimilarity between the different positions in Agios Vasilios cave (Two-way crossed SIMPER analysis results), indicated with grey color.** C, cave ceiling; L, left wall; R, right wall; ns, non-significant difference according to the results of PERMANOVA. For abbreviations of modalities see S2 Table.

| **(A) Sponge community structure** | | | | **(B) Sponge community function** | | | | |
|---|---|---|---|---|---|---|---|---|
| **Taxa** | **C vs L** | **C vs R** | **L vs R** | **Traits** | **Modalities** | **C vs L** | **C vs R** | **L vs R** |
| *Timea unistellata* | | | | Ecosystem engineering | Ec-Hf | | | |
| *Plakina bowerbankii* | | | | | Ec-Bi | | | |
| *Eurypon* sp. | | | | Maximum coverage | 10-30% | | | |
| *Penares euastrum* | | | | | 3-10% | | | |
| *Oscarella tuberculata* | | | | Feeding type | Ft-Ff | | | |
| *Hexadella pruvoti* | | | | Morphology (body design) | Mo-Ma | | | |
| *Raspaciona aculeata* | | | | | Mo-Tub | | | |
| *Hexadella racovitzai* | | | | Stratification | St-Up | | | |
| *Aplysina aerophoba* | | | | | St-Ba | | | |
| *Plakina trilopha* | | | | | St-In | | | |
| *Spirastrella cunctatrix* | | | | Sociability | So-M/C | | | |
| *Pleraplysilla spinifera* | | | | | | | | |
