# Supplementary figures and images for: Taxonomic and functional surrogates of sessile benthic diversity in Mediterranean marine caves
Source: PLoS One. 2017 Sep 6;12(9):e0183707. doi: 10.1371/journal.pone.0183707 (PMC5587111; doi:10.1371/journal.pone.0183707)

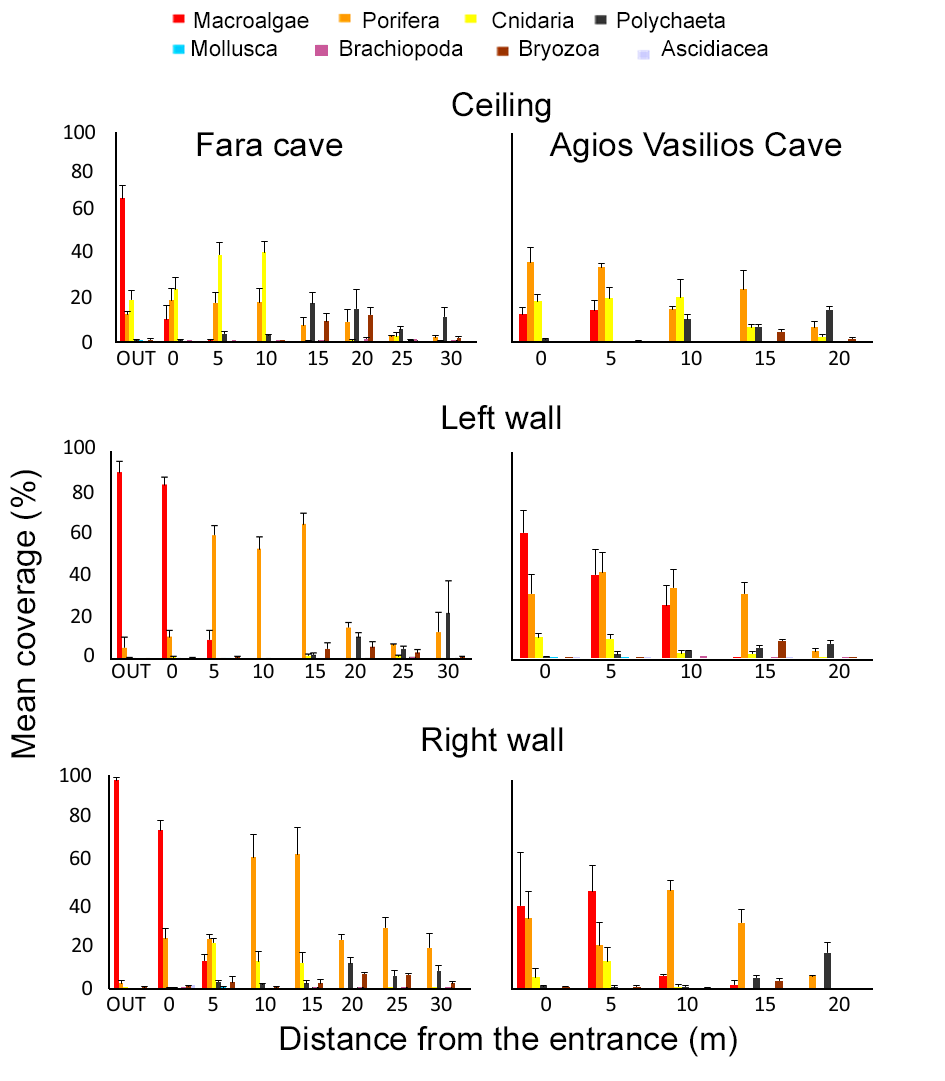

Supplement: S1 Fig — Standard error of mean (SE) is presented in error bars. (TIF) [file pone.0183707.s013.tif]
