# Supplementary material for: Taxonomic and functional surrogates of sessile benthic diversity in Mediterranean marine caves
Source: PLoS One. 2017 Sep 6;12(9):e0183707. doi: 10.1371/journal.pone.0183707 (PMC5587111; doi:10.1371/journal.pone.0183707)
Supplement: S4 Table — Summary of (A) taxa and (B) traits contributing by 50% to the calculated Bray-Curtis dissimilarity between the successive pairs of distance levels (5 m intervals) along the horizontal axis of Fara cave (Two-way crossed SIMPER analysis results), indicated with grey color. ns, non-significant difference according to the results of PERMANOVA. For abbreviations of modalities see S2 Table. (PDF) [file pone.0183707.s005.pdf]

S4 Table. Summary of (A) taxa and (B) traits contributing by 50% to the calculated Bray-Curtis dissimilarity between the successive pairs of distance levels (5 m intervals) along the horizontal axis of Fara cave (Two-way crossed SIMPER analysis results), indicated with grey color. ns, non-significant difference according to the results of PERMANOVA. For abbreviations of modalities see S2 Table.

(A) Total community structure

**Taxa**	**Out vs 0**	**0 vs 5**	**5 vs 10**	**10 vs 15**	**15 vs 20**	**20 vs 25**	**25 vs 30**
Turf-forming algae							ns
Palmophyllum crassum							ns
Encrusting Rhodophyta							ns
Acanthella acuta							ns
Raspaciona aculeata							ns
Hexadella pruvoti							ns
Haliclona mucosa							ns
Axinella damicornis							ns
Pleraplysilla spinifera							ns
Encrusting Bryozoa							ns
Ircinia variabilis							ns
Jaspis johnstoni							ns
Eurypon sp.							ns
Diplastrella bistellata							ns
Ircinia paucifilamentosa							ns
Oscarella microlobata							ns
Novocrania anomala							ns
Plakina bowerbankii							ns
Haliclona mucosa							ns
Spirastrella cunctatrix							ns
Agelas oroides							ns
Phorbas tenacior							ns
Madracis pharensis							ns
Dendroxea lenis							ns
Serpulidae							ns

(B) Total community function

**Traits**	**Modalities**	**Out vs 0**	**0 vs 5**	**5 vs 10**	**10 vs 15**	**15 vs 20**	**20 vs 25**	**25 vs 30**
Ecosystem engineering	Ec-Hf							ns
	Ec-Bo							ns
	Ec-Co							ns
	Ec-Bi							ns
Maximum coverage	10-30%							ns
	3-10%							ns
	0.3-1%							ns
	1-3%							ns
Feeding type	Ft-Ff							ns
	Ft-Pr							ns
	Ft-Sf							ns
Morphology (body design)	Mo-Fi							ns
	Mo-Tu							ns
	Mo-Ma							ns
	Mo-Ar							ns
	Mo-Nod							ns
	Mo-Tub							ns
	Mo-Ca							ns
Stratification	St-Up							ns
	St-Ba							ns
	St-In							ns
Sociability	So-Gr							ns
	So-So							ns
	So-M/C							ns
