# Supplementary material for: Taxonomic and functional surrogates of sessile benthic diversity in Mediterranean marine caves
Source: PLoS One. 2017 Sep 6;12(9):e0183707. doi: 10.1371/journal.pone.0183707 (PMC5587111; doi:10.1371/journal.pone.0183707)
Supplement: S8 Table — Summary of sponge (A) taxa and (B) traits contributing by 50% to the calculated Bray-Curtis dissimilarity between the successive pairs of distance levels (5 m intervals) along the horizontal axis of Fara cave (Two-way crossed SIMPER analysis results), indicated with grey color. ns, non-significant difference according to the results of PERMANOVA. For abbreviations of modalities see S2 Table. (PDF) [file pone.0183707.s009.pdf]

S8 Table. Summary of sponge (A) taxa and (B) traits contributing by 50% to the calculated Bray-Curtis dissimilarity between the successive pairs of distance levels (5 m intervals) along the horizontal axis of Fara cave (Two-way crossed SIMPER analysis results), indicated with grey color. ns, non-significant difference according to the results of PERMANOVA. For abbreviations of modalities see S2 Table.

(A) Sponge community structure

Taxa	Out vs 0	0 vs 5	5 vs 10	10 vs 15	15 vs 20	20 vs 25	25 vs 30
Pleraplysilla spinifera			ns				ns
Acanthella acuta			ns				ns
Penares euastrum			ns				ns
Raspaciona aculeata			ns				ns
Haliclona mucosa			ns				ns
Eurypon clavatum			ns				ns
Oscarella microlobata			ns				ns
Plakina bowerbankii			ns				ns
Hexadella pruvoti			ns				ns
Eurypon sp.			ns				ns
Diplastrella bistellata			ns				ns
Phorbas tenacior			ns				ns
Spirastrella cunctatrix			ns				ns
Agelas oroides			ns				ns
Dendroxea lenis			ns				ns

(B) Sponge community function

Traits	Modalities	Out vs 0	0 vs 5	5 vs 10	10 vs 15	15 vs 20	20 vs 25	25 vs 30
Ecosystem engineering	Ec-Hf			ns				ns
	Ec-Bo			ns				ns
	Ec-Bi			ns				ns
Maximum coverage	>30%			ns				ns
	10-30%			ns				ns
	3-10%			ns				ns
	1-3%			ns				ns
Feeding type	Ft-Ff			ns				ns
Morphology (body design)	Mo-En			ns				ns
	Mo-Tu			ns				ns
Stratification	St-Up			ns				ns
	St-Ba			ns				ns
	St-In			ns				ns
Sociability	So-M/C			ns				ns
